# Improving the management of Inherited Retinal Dystrophies by targeted sequencing of a population-specific gene panel

**DOI:** 10.1038/srep23910

**Published:** 2016-04-01

**Authors:** Nereida Bravo-Gil, Cristina Méndez-Vidal, Laura Romero-Pérez, María González-del Pozo, Enrique Rodríguez-de la Rúa, Joaquín Dopazo, Salud Borrego, Guillermo Antiñolo

**Affiliations:** 1 Department of Genetics, Reproduction and Fetal Medicine, Institute of Biomedicine of Seville, University Hospital Virgen del Rocío/CSIC/University of Seville, Seville, Spain; 2 Centre for Biomedical Network Research on Rare Diseases (CIBERER), Spain; 3 Department of Ophthalmology, University Hospital Virgen Macarena, Seville, Spain; 4 Computational Genomics Department, Centro de Investigación Príncipe Felipe (CIPF), Valencia, Spain; 5 Functional Genomics Node, (INB) at CIPF, Valencia, Spain

## Abstract

Next-generation sequencing (NGS) has overcome important limitations to the molecular diagnosis of Inherited Retinal Dystrophies (IRD) such as the high clinical and genetic heterogeneity and the overlapping phenotypes. The purpose of this study was the identification of the genetic defect in 32 Spanish families with different forms of IRD. With that aim, we implemented a custom NGS panel comprising 64 IRD-associated genes in our population, and three disease-associated intronic regions. A total of 37 pathogenic mutations (14 novels) were found in 73% of IRD patients ranging from 50% for autosomal dominant cases, 75% for syndromic cases, 83% for autosomal recessive cases, and 100% for X-linked cases. Additionally, unexpected phenotype-genotype correlations were found in 6 probands, which led to the refinement of their clinical diagnoses. Furthermore, intra- and interfamilial phenotypic variability was observed in two cases. Moreover, two cases unsuccessfully analysed by exome sequencing were resolved by applying this panel. Our results demonstrate that this hypothesis-free approach based on frequently mutated, population-specific loci is highly cost-efficient for the routine diagnosis of this heterogeneous condition and allows the unbiased analysis of a miscellaneous cohort. The molecular information found here has aid clinical diagnosis and has improved genetic counselling and patient management.

Inherited Retinal Dystrophies (IRD) are a heterogeneous group of diseases involving progressive degeneration of retinal photoreceptors. The most common form of IRD is Retinitis Pigmentosa (RP; MIM #268000), affecting 1 in ~4,000 individuals^1^. This condition is usually inherited as an autosomal recessive (ARRP), autosomal dominant (ADRP), X-linked recessive (XLRP) or sporadic/simplex trait (SRP), although rarer forms such as X-linked semi-dominant, mitochondrial and digenic have also been described^2^. Rod photoreceptors are the first cells affected in RP, followed by cones dysfunction in later stages of the disease. For this reason, other types of IRD as Cone-Rod dystrophies (CRD) and Leber Congenital Amaurosis (LCA), implicating both cones and rods photoreceptors, may present overlapping phenotypes with RP^3^ at their end-stages. Likewise, macular dystrophies as some cases of Stargardt disease (STGD) and Retinoschisis (RS) at atrophic stages can be difficult to diagnose at older ages. Additionally, there are syndromic forms of RP in which the disease is not limited to the eye, being Usher Syndrome (USH) and Bardet-Biedl Syndrome (BBS) the most prevalent of those disorders^1^,^2^.

This phenotypic overlap added to the high loci heterogeneity, with more than 200 IRD-associated genes and the fact that mutations in multiple genes can converge in the same phenotype^4^, make the clinical and molecular diagnosis of IRD an extremely difficult task. The recently introduced next-generation sequencing (NGS) technologies have shown to be very advantageous for the molecular characterization of IRD families^5^,^6^,^7^. Currently, targeted analysis of disease-specific candidate genes allows a better functional interpretation of sequence variations, and overcomes limitations of computational analysis related to the large amount of data generated by high-throughput sequencing platforms^6^,^8^,^9^. Despite clear improvements have been made, further optimization of current gene panels and bioinformatics pipelines implicating CNVs and other variants difficult to analyse, are needed to maximize diagnostic yield.

The prevalence of causative genes and mutations may vary considerably among different populations^10^. Thus, we developed a capture panel covering 64 retinal genes previously reported in IRD Spanish families. That panel was used to identify the genetic cause of the retinal disease in 32 unrelated Spanish families. Pathogenic mutations were found in 22 of the unsolved families (73%), whereas a conclusive diagnosis was achieved in 21 of them. That results in a diagnostic rate of 70%, demonstrating the validity of our genomic approach for genetic diagnosis of IRD.

## Results

### Clinical Assessment

The 32 index patients showed various clinical manifestations (see Supplementary Table S1). A total of 15 cases out of 32 presented night blindness as the first symptom, whereas the remaining families showed other initial symptoms such as decreased visual acuity or visual field constriction. Best corrected visual acuity (BCVA) of affected individuals ranged from 0.025 to 0.8. Moreover, age of onset and subsequent fundus appearance were different among families. All those clinical features together with the pedigree information, led to initially classify 10 families as arRP, 6 as adRP, 4 as USH, 4 as BBS, 2 as sRP, 2 as RS, 2 as STGD, 1 as XLRP and 1 as LCA. After the genetic findings, 6 cases were clinically reassessed.

### NGS data quality

A capture panel covering 64 retinal disease genes was used to identify the genetic cause of 32 IRD patients. On average, the mean coverage was 409× with 99% of reads covered >70× and 82% >200×. Different average coverages were achieved for the 64 targeted genes, ranging from 247× (*NRL*) to 565× (*BBS12*). Of note, the mean coverage for the hot spot exon ORF15 of *RPGR*, a region typically problematic for enrichment-based NGS methods, was average 389× and therefore suitable for variant calling. Application of variant calling, filtering and annotation of the sequencing data was performed as described above. This process concluded with an average of three candidate variants per sample to be validated and cosegregated by Sanger Sequencing. Mean output data for each step of the analysis pipeline are shown in Supplementary Fig. S1.

### Validation of the panel-based approach

For validation purposes, we used two previously solved families (RP15 and RP234), harbouring one and three known variants, respectively, as positive controls for our data analysis pipeline. These known mutations were correctly called and filtered, being the causal mutations highlighted after the bioinformatics analysis and demonstrating the diagnostic reliability of our approach. This is especially significant considering that one of the mutations of the positive control family RP234 was a Copy Number Variation (CNV) involving exon 12 of *EYS*^11^. In addition, the 21 variants previously detected by other techniques were also used to test the variant detection sensitivity of our study (Table 1). All these variants were also efficiently redetected by this platform, indicating a mutation detection rate of 100%.

### Identification of pathogenic mutations in unsolved families

**Figure 1:**
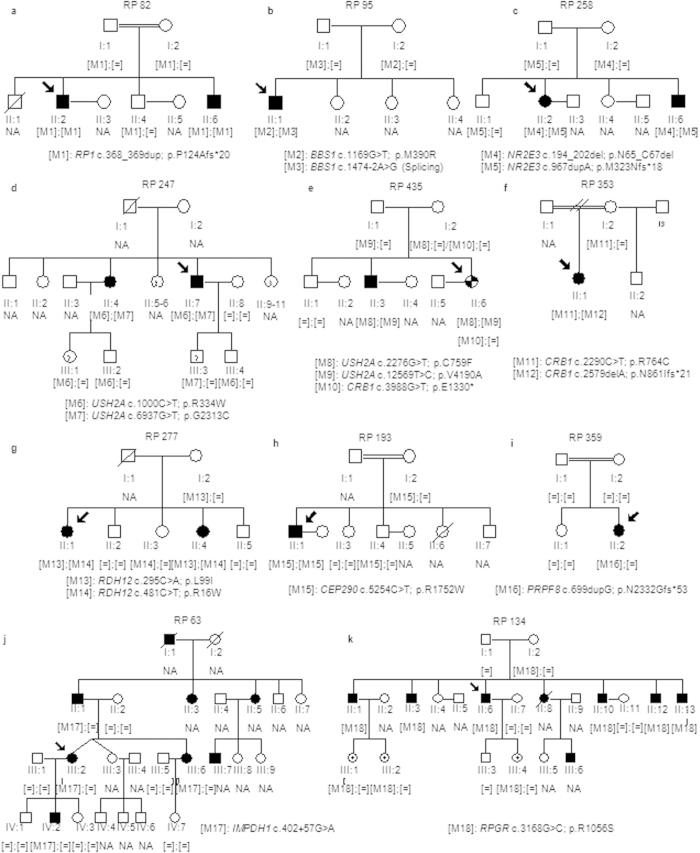
Segregation analysis of identified variants in the families with an initial clinical diagnosis of RP. (**a–d**) Pedigrees of arRP families. (**e**) Pedigrees of the family RP435. This family exhibited intrafamilial phenotypic variability. The phenotype of the index patient was more consistent with RP with early macular involvement (checkered symbol) while the diagnosis of arRP was confirmed in her affected brother (individual II:3) (solid symbol). (**f–h**) Pedigrees of families with a preliminary clinical diagnosis of arRP that has been reclassified to CRD. (**i**) Pedigree of an sporadic adRP family harboring a *de novo* frameshift mutation in *PRPF8*. (**j**) Pedigree of the adRP family RP63. (**k**) Pedigree of the partially dominant X-linked RP in which the oldest female carrier (II:8) showed clinical features of RP and the youngest (III:1, III:2 and III:4) had high myopia. Index patients are indicated with a black arrow. NA means non available DNA sample.

**Figure 2:**
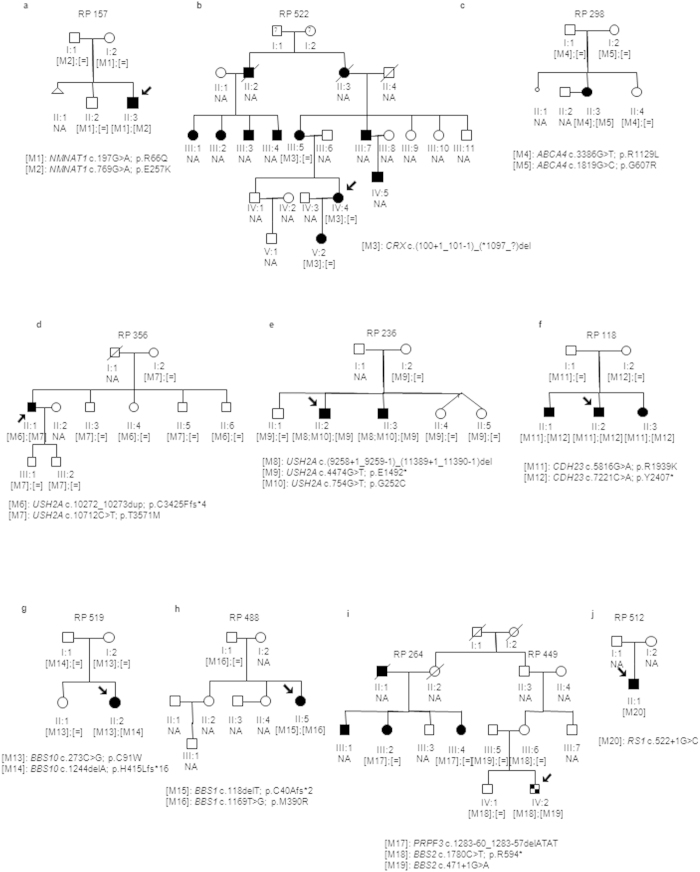
Segregation analysis of identified variants in the families with an initial clinical diagnosis of diverse retinal phenotypes. (**a**) Pedigree of the LCA family RP157. (**b**) Pedigree of the dominant LCA family RP522 harboring the heterozygous deletion of exons 3 and 4 in the *CRX* gene. (**c**) Pedigree of the STGD family RP298. (**d**–**f**) Pedigrees of the Usher Syndrome families. (**g**,**h**) Pedigrees of the Bardet-Biedl Syndrome families. (**i**) Pedigree of the related families RP264 and RP449. The index patient of the family RP449 (individual IV:2) received a clinical diagnosis of Bardet-Biedl Syndrome (checkered symbol) while the RP264 patients (II:4, III:4, III:5 and III:7) received a clinical diagnosis of adRP (solid symbols). (**j**) Pedigree of the retinoschisis family RP 512. Index patients are indicated with a black arrow. NA means non available DNA sample.

Sanger sequencing was performed to validate each variant and to carry out family cosegregation analyses. Moreover, when necessary, MLPA was performed to confirm presence of CNVs. These studies resulted in 37 potential pathogenic mutations in 22 out of the 30 families (73%) (Table 1; Figs 1 and 2). However, a full diagnosis was not achieved in one of these families (RP264) due to the nature of the detected mutation (Table 1), *in silico* predictions (Table 2) and the limited number of available family members for segregation analysis (Fig. 2), remaining a diagnostic rate of 70%. Fourteen of these variants were novel and absent in public databases as dbSNP, 1000 Genomes, EVS, ExAC and Database Genomic Variants (DGV), whereas 19 were previously reported as IRD causal mutations. Remarkably, a presumed pathogenic mutation in the dominant gene *CA4* (c.206G > A; p.Arg69His) was detected in the USH family RP236, but this variant did not conform to the recessive mode of inheritance of the family. Moreover, this mutation did not cosegregate with the disease since it was absent in affected individual II:3 while unaffected individuals I:2, II:4 and II:5 were heterozygous.

Detected candidate variants included 2 CNVs, 19 missense, 3 nonsense, 8 frameshift, 3 splicing and 2 intronic mutations. Novel frameshift and point mutations were absent in 200 matched controls sequenced by Sanger and in 267 healthy exomes from the Medical Genome Project^10^. The specific coverage for all detected variants is shown in Table 1 and *in silico* predictions in Table 2.

The most frequent mutated genes of this study were *USH2A* and *BBS1*, which appear in four and two of the solved cases, respectively. The rest of mutated genes were *ABCA4, BBS10, BBS2, CDH23, CEP290, CRB1, IMPDH1, NMNAT1, NR2E3, PRPF3, PRPF8, RDH12, RP1, RPGR, CRX* and *RS1*, all involved only in one family.

### Genotype-phenotype correlations and clinical refinements

The extreme phenotypic overlapping of IRD makes it difficult to establish clear clinical diagnoses. Most cases showed consistent results between clinical and genetic data, with the exception of families RP134, RP353, RP193, RP277 and RP522 (see Supplementary Table S1).

The mode of inheritance of family RP134 could not be assigned with complete confidence until the genetic diagnose was achieved. Our results showed that this family harboured a novel *RPGR* mutation that cosegregated in all members following a semidominant X-linked inheritance. Clinical reassessment showed that all female carriers presented visual impairments ranging from high myopia to mild RP, while males showed early onset disease with more severe symptoms. Families RP277 and RP353 had been diagnosed of arRP, but we suspected causal mutations in genes *CRB1* and *RDH12*, respectively. After reassessment, a diagnosis of LCA or early CRD was considered in both index patients. Affected member of family RP193 was diagnosed of arRP but the detection of a segregating homozygous mutation in *CEP290* led to the re-evaluation of the family, since mutations in this gene have been associated with severe ciliopathies such as BBS, Joubert syndrome, Meckel syndrome, Senior-Loken syndrome and LCA. The visual acuity decrease at 28 years old and the congenital nystagmus led to rectify the initial diagnosis of RP to a late onset form of LCA. Finally, affected individuals of family RP522 had received diverse clinical diagnoses, from retinoschisis to unspecified macular dystrophy and cone dystrophy. Targeted sequencing allowed us to detect a heterozygous CNV in *CRX*, a gene associated with dominant LCA, allowing a genetic diagnosis and clinical reclassification of this family.

### Phenotypic variability

The age of onset, the progression and the severity of the symptoms can vary greatly among affected individuals of retinal diseases even in the same family. Intrafamilial phenotypic variability was observed in family RP435 since index patient showed a severe form of IRD with early visual impairments, decreased visual field as first symptom and abolished ERG (see Supplementary Table S1), whereas affected individual II:3 showed a milder IRD with a later age of onset and slower disease progression. Two *USH2A* variants were detected in this family and both cosegregated with the disease in compound heterozygosity. However, those variants did not explain by themselves the early onset of symptoms of the index patient and the variations of phenotype between both affected members. Considering the presence of phenotype modifiers, we segregated the previously detected *CRB1* null mutation c.3988G > T in available family members (Fig. 1), confirming that the index patient carried three pathogenic alleles and individual II:3 only two in *USH2A*. Secondly, families RP95 and RP488 shared the *BBS1* mutation p.M390R which cosegregated with the disease in compound heterozygosity in both cases (Figs 1 and 2). However, family RP95 was diagnosed of sRP and family RP488 of BBS. Given that *BBS1* is a common BBS gene, family RP95 was subjected to clinical reassessment but no extraocular symptoms were detected, confirming non-syndromic RP in the index patient. Therefore, both families presented interfamilial variability caused by the same pathogenic allele.

## Discussion

For genetically and clinically heterogeneous diseases, where many different genes are involved without a major contribution of a single gene, parallel testing of several genes substantially increases the diagnostic yield and represents the most cost effective diagnostic choice^12^. In this study, we sequenced 32 IRD families using a customized panel covering 64 retinal genes. Reanalysis by gene panel sequencing of two of these families (RP258 and RP82), previously unsuccessfully studied by WES, led to the identification of disease-causing mutations in *RP1* (c.368_369dup; p.Pro124Alafs*20) and *NR2E3* (c.194_202del; p.Asn65_Cys67del and c.967dupA; p.Met323Asnfs*18). Although sequence data quality of WES was optimal, these variants were filtered out during bioinformatic analysis due to low coverage of these specific regions, supporting the fact that gene panel sequencing provides advantage over exome sequencing and should be the primary choice in a NGS-based diagnostic routine for highly heterogeneous disorders^6^.

Indeed, our panel-based next generation sequencing approach allowed us to detect all previously detected variants in the testing cohort yielding a diagnostic rate of 70% in a heterogeneous group of families with retinal degeneration. However, this percentage could be increased up to 73% if the pathogenicity of the intronic *PRPF3* variant is fully demonstrated. A total of 37 different mutations were found, 14 (37.8%) of which were novel variants newly associated with IRD in 9 genes that would have been missed by other standard approaches targeting only known alleles. The gene *USH2A* was more frequently affected than other genes (4 families), which is consistent with other recent reports^13^. However, we have to take into account that all our samples were previously tested and excluded for mutation in *EYS*, the most prevalent gene related to autosomal recessive retinal degeneration in our population^14^,^15^,^16^.

The selected group of patients included challenging cases for NGS data analysis such as simplex and autosomal dominant cases or cases with a poorly defined clinical picture. Remarkably, we found a possible dominant *de-novo* mutation in one case with sporadic RP. These results further support the role of this type of mutations, usually underestimated, in the development of simplex cases of RP which have major implications for genetic counselling of these families. In this light, our panel provided a high diagnostic yield regardless of the relatively small size of the panel (only 64 genes), mode of inheritance or clinical history. This unbiased strategy is particularly important for clinical genetics centres that can offer genetic diagnosis for a greater number of rare diseases even when the number of samples is small. The high yield may be the result of analyzing data for *de novo* mutations and CNVs that generally remain challenging despite the improvements to NGS technologies and CNV-detecting tools. This yield is comparable to other panels for the molecular diagnosis of retinal degeneration that simultaneously analyse a higher number of genes^17^. Although, the number of genes included in this panel will be higher as research evolves and more genes associated to IRD will be identified, these results indicate that increasing the number of genes in the panel may not substantially improve the diagnostic yield for a particular population. Those cases not resolved at this stage, may be investigated by applying clinical or whole exome sequencing.

CNVs are a significant event in the appearance of IRD but intrinsic methodology limitations commonly result in an underestimation of their allele frequencies^16^. Specific CNVs were detected in three patients: one involving exon 12 of *EYS* in a control patient, one in *CRX* (deletion of exons 3 and 4), a dominant LCA-associated gene, in a family (RP 522) with a poorly defined clinical history and another one in *USH2A* (deletion of exons 47–58) in an Usher patient (RP 236) who also presented two further *USH2A* variants and an adRP-associated mutation in *CA4* (c.206G > A; p.Arg69His)^18^. A toxic gain of function has been attributed to the *CA4* variant, leading to impaired trafficking of the CA4 protein, ER stress and apoptosis^19^. Nevertheless, this mutation neither conformed to the recessive mode of inheritance nor cosegregated with the disease in this family. In addition, we ruled out the possibility that this variant was acting as a modifier of the phenotype in this family since both affected siblings showed the same clinical features but only one harboured the *CA4* variant in heterozygous state. Those facts exclude the role of the *CA4* mutation in the IRD of family RP 236 and question its pathogenicity. These results suggest that the CNV found in *USH2A*, c.(9258 + 1_9259 − 1)_(11389 + 1_11390 − 1)del, in compound heterozygosity with the mutation c.4474G > T is the most likely cause of the disease.

The family RP193 harbouring the homozygous *CEP290* mutation c.5254C > T (p.R1752W), was initially diagnosed of arRP. This variant was previously reported by Eisenberger and coauthors as a third allele in a LCA family^20^. Here, the mutation c.5254C > T in the *CEP290* gene was found in association with a milder phenotype than typical LCA. The clinical results reported here are consistent with a previous study describing LCA patients with *CEP290* mutations^21^. Although most patients studied showed a severe visual acuity loss at first decade, they could observe a significant group of patients with atypical visual acuity preservation at later ages. Although more than 100 mutations in *CEP290* have been identified, no clear genotype-phenotype correlations have been established^22^. The results obtained in this study extend the phenotypic spectrum for *CEP290* mutations.

Six probands out of 22 individuals with positive molecular results showed inconsistent results between the molecular findings and their initial clinical diagnoses. Reassessment of the clinical information resulted in refinement of the clinical diagnoses to other retinal-related diseases or syndromes. This high clinical reclassifications rate (27%) is particularly important for the accurate diagnosis of this group of heterogeneous disorders. In addition, families showing clinical variability also complicate a proper diagnosis. Two families demonstrated phenotypic variability under the same mutant allele. The non-syndromic RP family RP95 and the BBS family RP488 harboured the common p.Met390Arg variant which causes 80% of BBS cases harboring mutations in BBS1^23^. Also, it has been reported that *BBS1* can cause either BBS or non-syndromic RP when mutated^24^. Therefore, our results confirmed those previous reports with two mutations in *BBS1* causing arRP. Moreover, a potential epistastic interaction was observed in family RP435. In addition to the identification of two mutations in *USH2A* as the most likely cause of the disease, data analysis and family segregation showed the presence of a heterozygous variant (c.3988G > T) in the gene *CRB1* only in patient II:6 and in her unaffected mother (I:2). The age of disease-onset was significantly different between the two affected siblings. While patient II:6, with the more severe phenotype (arRP with early macular involvement), presented the first symptoms shortly after birth, her affected sibling (II:3) showed a milder phenotype more typical of *USH2A*-associated mutations. We hypothesize that this phenomenon may be due to an epistatic effect of *USH2A* and *CRB1* mutations and may provide an explanation for the intrafamilial phenotypic variations observed in this family.

Analysis of intronic sequences flanking targeted exons showed two individuals harbouring low frequency variants in regions relatively distant (57–60 bp) from the natural donor and acceptor splice sites typically spanding intervals of 10 and 28 bases in length. Sequence variants affecting these sites or binding sites for splicing regulatory elements, which reside over a range of distances from the corresponding natural splice sites, have been shown to contribute to aberrant splicing and pathogenic phenotypes^25^. The intronic variant in *IMPDH1* was predicted to affect the splicing by creating a new donor-site according to MutationTaster tool. However, the intronic mutation of *PRPF3* was predicted to be benign by the *in silico* prediction tools used. In this regard, it has been reported that in some instances, functional non-canonical cryptic splicing sites can be challenging to identify by *in silico* prediction tools, because they use to be less well conserved than natural splice sites. In addition, these tools can occasionally provide conflicting results due to the use of different algorithms. Therefore, bioinformatic predictions should be complemented with additional studies to elucidate the pathogenic role of these variants in the RP of families 63 and 264. Despite this, the extensive cosegregation studies involving ten family members, the low MAF of the *IMPDH1* variant in public databases and its absence in 400 chromosomes of control population, let us to consider the *IMPDH1* mutation as likely causative in family RP 63.

In summary, although IRD panels including a larger number of genes have been successfully applied, we believe that sequencing diagnostic panels, targeting only genes and regions affecting a particular population, should represent the primary choice in a NGS-based diagnostic routing for highly heterogeneous disorders, followed by exome or genome sequencing of unsolved cases. Although research is in progress^26^, there is still no established treatment for hereditary blindness. The molecular findings reported here expand our understanding of retinal degeneration and greatly improve the genetic counselling management of our patient with IRD.

## Methods

### Sample collection

This study involved 208 Caucasian individuals belonging to 32 families selected on the basis of the phenotype and previous molecular genetics analyses. All affected individuals underwent a thorough ophthalmic evaluation as described elsewhere^27^ and were derived from the Ophthalmology Department to the Genetic, Reproduction and Fetal Medicine Department. Moreover, a group of 467 control individuals, which comprised unselected, unrelated race-, age-, and gender-matched Spanish individuals, was recruited. The research was conducted in accordance with the tenets of the Declaration of Helsinki^28^ and all experimental protocols were approved by the Institutional Review Boards of the University Hospital Virgen del Rocio (Spain). Written informed consent was obtained from all participants. Genomic DNA of all subjects was isolated from peripheral blood using standard procedures. DNA samples of index patients were processed for targeted sequencing.

### Previous genetic analyses

DNA sample from index patients was first analysed by multiple approaches for the identification of causative variants, including an available microarray analysis (Asper Biotech, Tartu, Estonia), a custom genome resequencing microarray^11^, whole exome sequencing and direct sequencing^15^ and multiplex ligation dependent probe amplification (MLPA)^16^ of *EYS.* Pathogenic variants detected by these techniques are marked in Table 1. Of note, no candidate causal mutations were found in two of the families sequenced by whole exome sequencing (RP82 and RP258).

Two of the IRD families included in this study (RP15 and RP234) were previously solved by these previous techniques and were used as positive controls. The remaining thirty families were still unsolved. Among them, 12 were found to carry 21 likely disease causing mutations detected by the above methods (Table 1).

### Design of the capture panel

The detection rate by targeted sequencing is substantially affected by population-specific mutation frequencies. Therefore, in order to select the most appropriate retinal genes for our population, we collected all mutated genes of our local cohort and performed an extensive literature review of genes with pathogenic mutation in Spanish population. This search was conducted using the following terms in PubMed: (Spain) AND (Retinal Dystrophies OR Retinal Degeneration OR Ciliopathies). These selection criteria resulted in 64 retinal genes (see Supplementary Table S2).

A custom IRD panel containing all coding exons plus 25 bp of intronic flanking sequences of the 64 selected genes was developed using SureDesign (www.agilent.com/genomics/suredesign). Moreover, deep intronic sequences of *USH2A*, *CEP290* and *OFD1* genes, containing prevalent pathogenic mutations, were also included (see Supplementary Table S2). The probes covered a total of 1106 exons with a total size of 311.683 Kbp.

### Library preparation and targeted sequencing

The in-solution target enrichment was performed according to Agilent’s standard protocol for Illumina library preparation (SureSelect^XT^ Target Enrichment System for Illumina Paired-end Sequencing Library) following the manufacturer’s instructions. Briefly, sheared DNA was hybridized with specific probes, and captured fragments were separated using streptavidin-coated magnetic beads and buffers. The selected regions were then PCR-amplified using Illumina PCR primers. Illumina libraries were quantified using the Agilent 2100 Bioanalyzer (Agilent Technologies), qPCR and spectrophotometric measurements and the final products were sequenced in a MiSeq instrument using a v2 (300 cycles) reagent kit (Illumina).

### Sequence data analysis

In order to identify causative mutations in the sequenced samples, we used our validated data analysis pipeline^29^ with some modifications. Sequence reads were mapped against the human genome reference (hg19) using SureCall software (Agilent, version 2.1). The coverage and the percentage of reads on target were analysed using the BEDtools package. SNVs and indels were called and filtered using GATK software (version 1.4)^30^. Filtered variants with a coverage >20× were annotated using Annovar^31^ and those with a MAF higher than 0.01 in at least one of the searchable databases (dbSNP, 1000 Genomes, Exome Variant Server (EVS), Exome Aggregation Consortium (ExAC) and an in-house developed database containing exome sequences from 267 control individuals^32^) were excluded. To detect known disease-associated mutations, the remaining variants were compared to human mutation databases such as HGMD and ClinVar using the TEAM tool (http://team.babelomics.org)^33^. The prioritization was performed using the BiERapp tool (http://bierapp.babelomics.org)^34^, generating a list of candidate causative mutations.

Potential copy number variations (CNV) were identified by Surecall (Agilent) on mapped reads using the “pair analysis” option. Coverage of each target region of the sample of interest was internally compared to data of other samples of the same run. Deletions and duplications were reported if they were detected against at least >50% of samples and had a score >0.7. Identified CNVs were compared with structural variations present in the Database of Genomic Variants (DGV).

### Pathogenicity assessment and validation of candidate variants

Sanger sequencing was used to validate and cosegregate candidate variants in available family members. Likewise, a screening of the novel changes in 200 matched control individuals was also carried out by direct sequencing. Additionally, the presence of all candidate variants were checked in the above mentioned database containing the exomes from 267 healthy controls of the Spanish population (The Medical Genome project, http://www.medicalgenomeproject.com/). The pathogenicity of novel SNVs was predicted by PolyPhen-2 (http://genetics.bwh.harvard.edu/pph2/) and SIFT (http://sift.bii.a-star.edu.sg) algorithms. Additionally, to estimate the effect of intronic mutations on the splicing process we used NNSPLICE (http://www.fruitfly.org/seq_tools/splice.html), Mutation Taster (http://www.mutationtaster.org/) and Human Splicing Finder (HSF; http://www.umd.be/HSF). The nomenclature of variants was adjusted to the Human Genome Variation Society (http://www.hgvs.org) guidelines using Mutalyzer (http://www.LOVD.nl/mutalyzer).

CNVs were validated by multiplex ligation dependent probe amplification (MLPA). SALSA MLPA P361, P362 and P221 were employed to analyse large rearrangements following manufacturer´s recommendations (MRC Holland, Amsterdam, The Netherlands).

## Additional Information

**How to cite this article**: Bravo-Gil, N. *et al.* Improving the management of Inherited Retinal Dystrophies by targeted sequencing of a population-specific gene panel. *Sci. Rep.* **6**, 23910; doi: 10.1038/srep23910 (2016).

## Supplementary Material

Supplementary Table S1

Supplementary Figure S1

Supplementary Table S2

## Figures and Tables

**Table 1:** Causative variants detected in all solved families.

Fam (RP)	Gene	Nt Change	Prot Change	Status	Cvg Mut	Ref.
134	*RPGR*	c.3168G > C	p.R1056S	Hem	320	Novel
234^#^	*EYS*	c.1971delT (*)	p.S658Vfs*4 (*)	Hem	360	11
		c.(1766 + 1_1767 − 1)_(2023 + 1_2024 − 1)del (*)	NA	Het	–	
258	*NR2E3*	c.194_202del	p.N65_C67del	Het	136	35
		c.967dupA	p.M323Nfs*18	Het	400	Novel
298	*ABCA4*	c.3386G > T (*)	p.R1129L (*)	Het	392	36
		c.1819G > C	p.G607R	Het	443	37
356	*USH2A*	c.10272_10273dupA (*)	p.C3425Ffs*4 (*)	Het	739	38
		c.10712C > T	p.T3571M	Het	721	
435	*USH2A*	c.2276G > T (*)	p.C759F (*)	Het	899	39
		c.12569T > C	p.V4190A	Het	813	Novel
	*CRB1*	c.3988G > T (*)	p.E1330* (*)	Het	738	40
15^#^	*RPGR*	c.2405-2406delAG (*)	p.E802Gfs*32 (*)	Hem	174	41
95	*BBS1*	c.1169T > G	p.M390R	Het	401	23
		c.1474-2A > G	NA	Het	277	Novel
353	*CRB1*	c.2290C > T (*)	p.R764C (*)	Het	699	42
		c.2579delA	p.N861Ifs*21	Het	860	Novel
359	*PRPF8*	c.6994dupG	p.D2332Gfs*53	Het	363	Novel
488	*BBS1*	c.1169T > G (*)	p.M390R (*)	Het	629	23
		c.118delT	p.C40Afs*2	Het	676	Novel
512	*RS1*	c.522 + 1G > C	NA	Hem	289	Novel
63	*IMPDH1*	c.402 + 57G > A	NA	Het	139	43
82	*RP1*	c.368_369dup	p.P124Afs*20	Hom	269	44
118	*CDH23*	c.5816G > A	p.R1939K	Het	169	Novel
		c.7221C > A	p.Y2407*	Het	180	45
157	*NMNAT1*	c.197G > A	p.R66Q	Het	467	ExAC
		c.769G > A	p.E257K	Het	421	46
193	*CEP290*	c.5254C > T	p.R1752W	Hom	173	47
236	*USH2A*	c.754G > T	p.G252C	Het	346	Novel
		c.4474G > T	p.E1492*	Het	329	48
		c.(9258 + 1_9259 − 1)_(11389 + 1_11390 − 1)del	NA	Het	–	Novel
247	*USH2A*	c.6937G > T	p.G2313C	Het	294	Rs1
		c.1000C > T (*)	p.R334W (*)	Het	304	49
264	*PRPF3*	c.1283-60_1283-57delATAT	NA	Het	29	Rs2
277	*RDH12*	c.295C > A (*)	p.L99I (*)	Het	292	50
		c.481C > T	p.R161W	Het	313	51
449	*BBS2*	c.1780C > T	p.R594*	Het	166	Novel
		c.471 + 1G > A	NA	Het	147	Novel
519	*BBS10*	c.1244delA	p.H415Lfs*16	Het	258	52
		c.273C > G	p.C91W	Het	283	53
522	*CRX*	c.(100 + 1_101 − 1)_(*1097_?)del	NA	Het	–	Novel

Cvg Mut: coverage for each position. Asterisks “(*)”: variants previously detected by other techniques. Pads

“#”: families solved prior to this study. Rs1: rs199840367 and rs2: rs66790680. ExAC: Variant present in the Exome Aggregation Consortium.

**Table 2:** *In silico* predictions for missense and splicing novel and non disease-associated mutations.

Family (RP)	Gene	Mutation	Polyphen (score)	Sift (score)	NNSPLICE	HSF	Mutation taster
134	*RPGR*	p.R1056S	Benign (0.347)	Damaging (0.02)	–	–	
435	*USH2A*	p.V4190A	Damaging (1)	Damaging (0)	–	–	
95	*BBS1*	c.1474-2A > G	–	–	Acceptor-site broken	Acceptor-site broken	Acceptor-site broken
512	*RS1*	c.522 + 1G > C	–	–	Donor-site broken	Probably no impact	Donor-site broken
63	*IMPDH1*	c.402 + 57G > A	–	–	Probably no impact	Probably no impact	Donor-site gained
118	*CDH23*	p.R1939K	Damaging (0.997 )	Tolerated (0.56)	–	–	
157	*NMNAT1*	p.R66Q	Damaging (0.962)	Damaging (0)	–	–	
193	*CEP290*	p.R1752W	Damaging (1)	Damaging (0.01)	–	–	
236	*USH2A*	p.G252C	Damaging (0.999)	Damaging (0.01)	–	–	
247	*USH2A*	p.G2313C	Damaging (1)	Damaging (0)	–	–	
264	*PRPF3*	c.1283-60_1283-57del	–	–	Probably no impact	Probably no impact	Probably no impact
449	*BBS2*	c.471 + 1G > A	–	–	Donor-site broken	Donor-site broken	Donor-site broken
