# Supplementary material for: Improving the management of Inherited Retinal Dystrophies by targeted sequencing of a population-specific gene panel
Source: Sci Rep. 2016 Apr 1;6:23910. doi: 10.1038/srep23910 (PMC4817143; doi:10.1038/srep23910)

SUPPLEMENTARY INFORMATION

Improving the management of Inherited Retinal Dystrophies by targeted sequencing of a population-specific gene panel.

Nereida Bravo-Gil[#], Cristina Méndez-Vidal[#], Laura Romero-Pérez, María González-del Pozo, Enrique Rodríguez-de la Rúa, Joaquín Dopazo, Salud Borrego and Guillermo Antiñolo[*].

# Equally contributing authors

* Corresponding author

Supplementary figure S1. Mean output data for each step of bioinformatic analysis. Total variants: All variants generated by the variant-calling. Quality filtering: variants with a coverage >20X, a percentage of reads supporting the variant allele >25% and with a disequilibrium between number of forward and reverse sequences <15%. Recurrence filtering: variants with an allelic frequency <0.01 in the searchable databases. Exonic variants: variants located in coding exons and UTRs. Candidate variants: Nonsynonymous variants consistent with the inheritance mode. Cosegregated variants: variants confirmed by Sanger sequencing that cosegregated with the disease in all family members.

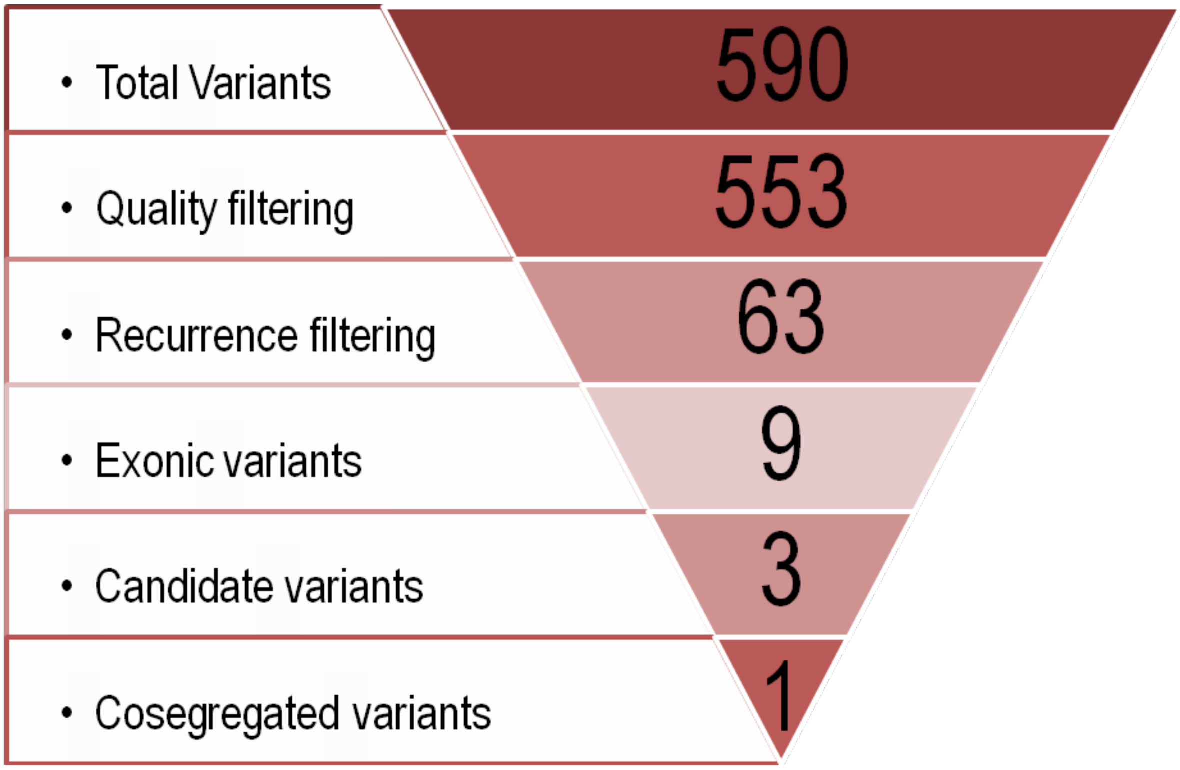

Supplement: Supplementary Figure S1 [file srep23910-s2.pdf]
